# Seed-Mediated Synthesis of Thin Gold Nanoplates with Tunable Edge Lengths and Optical Properties

**DOI:** 10.3390/nano13040711

**Published:** 2023-02-13

**Authors:** Zhun Qiao, Xinyu Wei, Hongpo Liu, Kai Liu, Chuanbo Gao

**Affiliations:** Frontier Institute of Science and Technology, Xi’an Jiaotong University, Xi’an 710054, China

**Keywords:** Au nanoplates, thin nanoplates, seed-mediated synthesis, size engineering, surface-enhanced Raman scatting

## Abstract

Thin Au nanoplates show intriguing localized surface plasmon resonance (LSPR) properties with potential applications in various fields. The conventional synthesis of Au nanoplates usually involves the formation of spherical nanoparticles or produces nanoplates with large thicknesses. Herein, we demonstrate a synthesis of uniform thin Au nanoplates by using Au–Ag alloy nanoframes obtained by the galvanic replacement of Ag nanoplates with HAuCl_4_ as the seeds and a sulfite (SO_3_^2−^) as a ligand. The SO_3_^2−^ ligand not only complexes with the Au salt for the controlled reduction kinetics but also strongly adsorbs on Au {111} facets for effectively constraining the crystal growth on both basal sides of the Au nanoplates for controlled shape and reduced thicknesses. This seed-mediated synthesis affords Au nanoplates with a thickness of only 7.5 nm, although the thickness increases with the edge length. The edge length can be customizable in a range of 48–167 nm, leading to tunable LSPR bands in the range of 600–1000 nm. These thin Au nanoplates are applicable not only to surface-enhanced Raman spectroscopy with enhanced sensitivity and reliability but also to a broader range of LSPR-based applications.

## 1. Introduction

Plasmonic nanocrystals, especially those of Ag and Au, have attracted intensive interest in recent years owing to their prominent localized surface plasmon resonance (LSPR) properties and, as a result, they have been applied broadly in surface-enhanced Raman spectroscopy (SERS) [1,2,3], biosensing [4,5,6,7], imaging [5,8,9], and catalysis [8,10,11]. Because the LSPR properties depend heavily on the size and shape of these nanocrystals, it is highly desirable to achieve customizable sizes and shapes toward tunable LSPR bands in the UV–visible near infrared (NIR) region, according to the needs of applications. To date, significant efforts have been devoted to the shape control of Ag and Au nanocrystals, leading to the convenient availability of nanocrystals with diverse shapes, such as spheres [12,13], polyhedra [14,15,16], bipyramids [17,18], rods [1,19,20], wires [21,22], and plates [23,24,25,26,27,28,29]. However, the synthesizing of nanocrystals with specific shapes, e.g., thin Au nanoplates, still needs substantial perfecting in terms of the morphological yield and size controllability.

Thin Au nanoplates are important types of plasmonic nanocrystal. Compared with Ag, Au nanoplates show superior resistance to chemical oxidation. The anisotropic shapes of Au nanoplates shift the LSPR to long wavelengths in the visible and NIR regions, well apart from the interband electron-transition region (<450 nm), enabling excellent Ag-like LSPR properties [4,6,9,30]. The synthesis of Au nanoplates dates back to two decades ago, when Liz-Marzán et al. reported the first plate-like Au nanostructure obtained by a chemical-reduction method [31]. Unfortunately, these seedless syntheses usually produce Au nanoplates with many side products, including nanospheres and nanorods [32,33,34,35]. This could be attributed to the difficulty in controlling the crystal structures of self-nucleated seeds, among which only those with planar defects eventually evolve into nanoplates [36]. Mirkin et al. developed an approach that involved preparing Au nanoplates with pre-synthesized Au seeds, obtained by rapidly reducing HAuCl_4_ by NaBH_4_, in the synthesis [25,37,38]. The preferential adsorption of iodide, either as an impurity in cetyltrimethylammonium bromide (CTAB) or added purposely, on Au {111} facets plays an important role in the formation of Au nanoplates [39]. However, the product is also a mixture of nanoplates and spherical nanoparticles [39,40,41,42], because the Au seeds are still mixed nanoparticles with various crystal structures [43]. Therefore, these prior syntheses usually require a laborious purification process to obtain Au nanoplates with satisfactory morphological purity [42].

A few recent works demonstrated the improved synthesis of Au nanoplates by introducing additional control over the seed structures. Some oxidizing agents, such as H_2_O_2_ and tri-iodide ions (I_3_^−^), were found to be effective in removing undesirable self-nucleated Au seeds without planar defects in a seedless chemical-reduction synthesis, giving rise to Au nanoplates with a high yield [24,26]. Pre-synthesized metal seeds with exclusive planar defects, e.g., defected Au–Ag nanoframes, were also introduced into a seed-mediated synthesis, which produced Au nanoplates, each with a central hole, with a high yield [44]. However, the Au nanoplates from these syntheses usually possess relatively large thicknesses, typically > 15 nm, even for those with sub-100-nanometer edge lengths. This leads to low aspect ratios and considerably large fractions of light scattering in the overall extinction spectra, which may impose undesirable influences on their plasmonic applications. Moreover, the size tunability of Au nanoplates is still not satisfactory. Therefore, there is still a significant need to develop alternative strategies for optimizing the synthesis of Au nanoplates.

Herein, we report a robust seed-mediated synthesis of Au nanoplates with the advantages of decreased thickness, high morphological yield, customizable edge lengths, and tunable optical properties. We chose Au–Ag-alloy nanoframes prepared by the galvanic replacement of Ag nanoplates with HAuCl_4_ with retained planar defects as the seeds to satisfy the symmetry-breaking requirement for the formation of the plate-like nanostructure. Sulfite (SO_3_^2−^) played a key role in the subsequent growth of thin Au nanoplates by coordinating with the Au salt and capping on the Au {111} surface. While the former afforded Au(SO_3_)_2_^3−^ a low standard reduction potential (*E*^o^ = 0.11 V) to reliably prevent the destructive etching of the Au–Ag-alloy nanoframes [45] and self-nucleation events [30,46], the latter proved effective in regulating the nanoplate formation and confining the crystal growth to reduced thicknesses. As a result, the Au nanoplates were reproducibly synthesized with controllable edge lengths (48–167 nm), thicknesses (7.5–22.5 nm, depending on the edge length), and tunable LSPR bands in the range of 600–1000 nm. Thanks to their high chemical stability and outstanding optical properties, these Au nanoplates demonstrated excellent sensitivity in detecting molecules of interest by SERS under harsh conditions. Because the LSPR bands of Au nanoplates are located in the near-infrared optical window (NIR-I) of biological tissues, we also expect these materials to be applied in photothermal therapy for tumors [47,48,49]. Therefore, this work paves an attractive path towards the synthesis of thin Au nanoplates with designable optical properties for many LSPR-based applications.

## 2. Materials and Methods

Synthesis of Ag nanoplates. The Ag nanoplates were synthesized by using a chemical-reduction method [50]. In a typical synthesis, 0.2 mL of AgNO_3_ (0.1 M), 12 mL of trisodium citrate (0.075 M), and 0.48 mL of H_2_O_2_ (30 wt%) were dissolved in 200 mL of H_2_O. Into this solution was injected 1.2 mL of freshly made NaBH_4_ (0.1 M) under vigorous stirring. The solution quickly turned blue and was collected as a stock solution after 30 min.

Synthesis of Au–Ag-alloy nanoframes. The Au—Ag-alloy nanoframes were synthesized by the galvanic replacement reaction of Ag nanoplates with HAuCl_4_. In a typical synthesis, the Ag nanoplates were recovered by centrifugation from 20 mL of the stock solution and redispersed in 3 mL of H_2_O. Next, 2.0 mL of HAuCl_4_ (0.1 mM) was slowly added to the solution of the Ag nanoplates using a syringe pump at a rate of 5 mL h^−1^ under vigorous stirring. The resulting Au–Ag-alloy nanoframes were collected by centrifugation and washed with H_2_O.

Preparation of growth solution of Au. A sulfite-coordinated Au precursor, i.e., Na_3_Au(SO_3_)_2_, was prepared as the growth solution by following our previously reported protocol [30,46]. Typically, 40 μL of HAuCl_4_ (0.25 M), 240 μL of NaOH (0.2 M), and 3.00 mL of Na_2_SO_3_ (0.01 M) were dissolved in 4.72 mL of H_2_O. The solution was left undisturbed overnight before use.

Synthesis of Au nanoplates. The pre-synthesized Au nanoframes were suspended in 2 mL of H_2_O. To this solution were added 6.72 mL H_2_O, 500 µL of polyvinylpyrrolidone (PVP, 5 wt%, Mw 10000), 60 μL L-ascorbic acid (AA) (0.1 M), 120 μL NaOH (0.1 M), and 0.6 mL of the growth solution of Au. The reaction was then left undisturbed at 30 °C for 12 h. Finally, Au nanoplates (edge length, ~48 nm) were collected by centrifugation, washed with H_2_O, and redispersed in 2 mL of H_2_O.

Synthesis of Au nanoplates with different edge lengths. The Ag nanoplates of various sizes were synthesized by a previously reported seed-mediated growth method [50]. The Au–Ag-alloy nanoframes of different sizes were synthesized with these Ag nanoplates. These Au–Ag-alloy nanoframes were then used as the seeds for the synthesis of Au nanoplates following a similar procedure to that described above.

SERS analysis with the Au-nanoplate substrate in oxidative environments. Typically, 25 μL of Au nanoplates (~48 nm) were dried on a clean silicon wafer (8 mm × 8 mm). Next, a specific amount of an aqueous crystal violet solution (10^−6^ M, with additional 2 mM Fe(NO_3_)_3_ or 10 mM H_2_O_2_ when oxidative species were involved) was dropped and dried on the substrate. The substrate was then washed with H_2_O to remove free-crystal-violet molecules. Raman spectra were then recorded from the substrate with a 633-nanometer He–Ne laser line at room temperature. For all measurements, the laser power was 3 mW and the signal-acquisition time was fixed at 10 s.

Characterizations. Transmission electron microscopy (TEM) was performed with a Hitachi HT-7700 electron microscope equipped with a tungsten filament at an accelerating voltage of 120 kV. High-resolution TEM (HRTEM) and energy-dispersive X-ray spectroscopy (EDS) was performed on a Philips Tecnai F20 FEG-TEM at an accelerating voltage of 200 kV. The UV–Vis spectra were measured on an Ocean Optics HR2000+ES spectrophotometer. Atomic-force microscopy (AFM) was conducted on Cypher through the tapping mode. Elemental analysis was conducted by inductively coupled plasma mass spectrometry (ICP-MS) on an Agilent 7500CE. Raman spectra were collected with a LabRAM HR800 confocal Raman spectrophotometer equipped with a 633-nanometer He–Ne laser.

## 3. Results and Discussion

**Figure 1:**
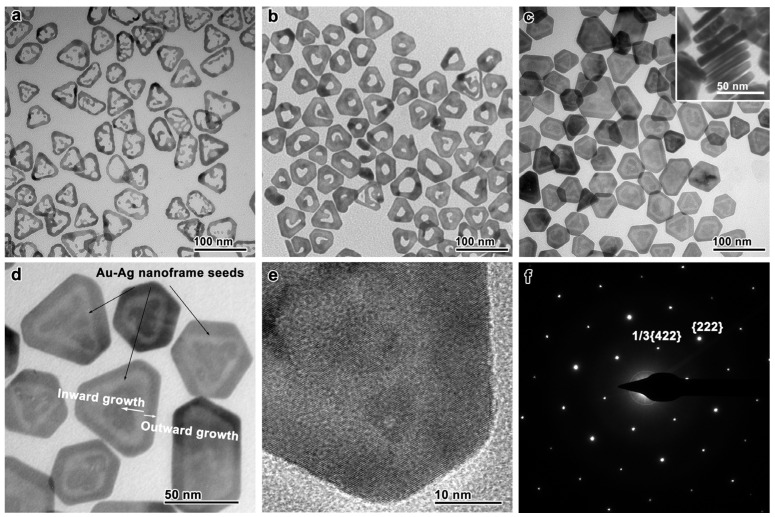
Monitoring the synthesis of Au nanoplates by TEM imaging. (**a**) Au–Ag-alloy nanoframes as the seeds for the seed-mediated synthesis. (**b**) An intermediate of the crystal growth. (**c**) The final product of the Au nanoplates. Inset: Au nanoplates standing vertically. (**d**) An enlarged TEM image, showing the low-contrast “watermark” corresponding to the Au–Ag-alloy-nanoframe seed and the schematic directions of the seed-mediated growth. (**e**) HRTEM image of the Au nanoplates. (**f**) The electron-diffraction pattern of the Au nanoplates.

The seed-mediated synthesis of Au nanoplates requires pre-synthesized seeds with multiple planar defects [33,36]. Such planar defects can be found in Ag nanoplates, which are obtainable in a high yield by a well-established synthesis protocol [23]. When these Ag nanoplates were directly used as the seeds, the resulting Au nanoplates were essentially core-shell nanostructures [30] or nanoframes if a galvanic replacement reaction was involved [51]. In order to achieve the well-controlled synthesis of Au nanoplates with minimal Ag component, these Ag nanoplates (edge length, ~45 nm; thickness, ~6.8 nm; TEM, Figure S1) were converted into Au–Ag-alloy nanoframes through a galvanic replacement reaction with HAuCl_4_ prior to the seed-mediated synthesis (Figure 1a; TEM, Figure S2; EDS mapping, Figure S3). In this reaction, Au preferentially grew on the edges of the Ag nanoplates, accompanying an oxidative etching of the Ag cores, leading to a hollow nanostructure. These nanoframes retained the original planar defects in the Ag nanoplates, as evidenced by the formation of many anisotropic nanostructures through the crystal growth on these seeds [44,45,52]. The planar defects clearly satisfied the symmetry-breaking requirement for the growth of Au nanoplates.

The Au nanoplates were synthesized by controlled crystal growth on the Au–Ag-alloy nanoframes. A growth solution of Au was first prepared by mixing sodium hydroxide (NaOH), sodium sulfite (Na_2_SO_3_), and chloroauric acid (HAuCl_4_) in water, which was left undisturbed overnight [30,46]. To this growth solution were added the Au–Ag-alloy nanoframes, PVP, L-AA, and NaOH to initiate the seed-mediated growth. After the reaction system was kept at 30 °C for 12 h, the Au nanoplates were collected by centrifugation and washed with water. Figure 1b shows the TEM image of the intermediate collected during the reaction. By comparing it with the TEM image of the Au–Ag-alloy nanoframes (Figure 1a), one can infer that the Au growth started from the nanoframes and proceeded inward to fill the inner cavity of the nanoframes. As a result, each nanoplate possessed a single hole in its center. After the extensive crystal growth, the Au fully filled the inner cavity of the nanoframes, forming Au nanoplates that resembled the shapes of the Au–Ag-alloy nanoframes or the original Ag nanoplates (Figure 1c and Figures S4 and S5). A triangular “watermark”-like pattern with a brighter image contrast was found in each Au nanoplate, corresponding to the Au–Ag-alloy-nanoframe seed (Figure 1d). The brighter image contrast can be attributed to the presence of Ag, which possesses a smaller atomic number than Au, in these positions. By comparing the shapes of the watermark pattern and the final Au nanoplate, one can conclude that the Au not only grew inward to fill the inner cavity of the Au–Ag-alloy nanoframe, but also grew outward to reshape the Au nanoplate into truncated triangular or hexagonal shapes.

The coordination of the HAuCl_4_ with SO_3_^2−^ greatly reduced its reduction potential, which ruled out the destructive etching of the Au–Ag-alloy nanoframes by the Au salt. In addition, the coordination decreased the reduction rate of the Au salt and, thus, the growth rate of the nanocrystals. As a result, Au nanoplates with well-defined shapes were been synthesized (average edge length, ~48 nm). No self-nucleated side products, such as spherical nanoparticles, were formed in this process. The average thickness was estimated to be ~8 nm according to the TEM image of the nanoplates standing vertically on the grid (Figure 1c, inset). The thickness was close to that of the original Ag nanoplates (Figure S1), suggesting that the Au growth was mainly in the lateral directions. Thus, the Au growth on the basal sides was greatly restrained, which can be attributed to the strong adsorption of SO_3_^2−^ on the basal Au {111} facets, highlighting the superiority of our design of the synthesis to those used in many previous strategies.

The structure of the Au nanoplates was further verified by HRTEM imaging and electron diffraction (Figure 1e,f). The HRTEM image showed clear lattices corresponding to the [111]-zone axis. The formally forbidden 1/3{422} diffractions were observable in the electron-diffraction pattern, which originated from the stacking faults along the <111> direction. These results suggest that the crystal structures of the original Ag nanoplates (i.e., {111} facets and abundant stacking faults) were successfully inherited by the Au nanoplates through the seed-mediated crystal growth.

**Figure 2:**
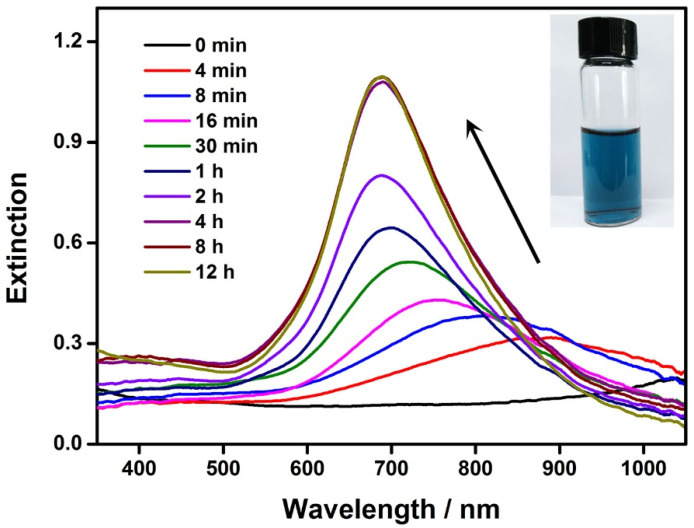
Evolution of the LSPR during the growth of the Au nanoplates. The UV–Vis spectra were recorded at varying reaction times. Inset: a photograph of the Au nanoplates.

The seed-mediated growth of the Au nanoplates was monitored by UV–Vis spectroscopy (Figure 2). The initial reaction solution (i.e., reaction time, 0 min) appeared near-colorless because the LSPR band of the Au–Ag-alloy nanoframes moved to the infrared region of the spectrum. During the growth of the Au on these seeds, a progressive blueshift of the LSPR was observed, corresponding to the continuous decrease in the size of the inner cavity in the nanoframes. Accompanying the shift in the LSPR band position, its intensity underwent a continuous increase. This can be attributed to the volume expansion of the Au–Ag nanoframes during their growth into nanoplates because the extinction cross-section was proportional to the volume of the nanocrystals, according to the Mie theory [53]. After 8 h of crystal growth, the LSPR band of the Au nanoplates became stable in terms of both the band position and the intensity, indicating the end of the crystal growth. The final solution of the Au nanoplates showed a deep blue color, which was consistent with the LSPR band eventually shifting to a wavelength of 685 nm (Figure 2, inset). It is worth noting that only an in-plane dipolar LSPR band was observed in the UV–Vis spectrum. This is because in-plane quadrupolar LSPR becomes obvious only when the Au nanoplates are sufficiently large and the shapes and sizes are highly uniform. Our experiment produced Au nanoplates with a small average edge length of 48 nm. In addition, they showed variations in both shape and edge length. We expect well-resolved quadrupolar LSPR bands from Au nanoplates with even larger sizes and improved uniformity.

**Figure 3:**
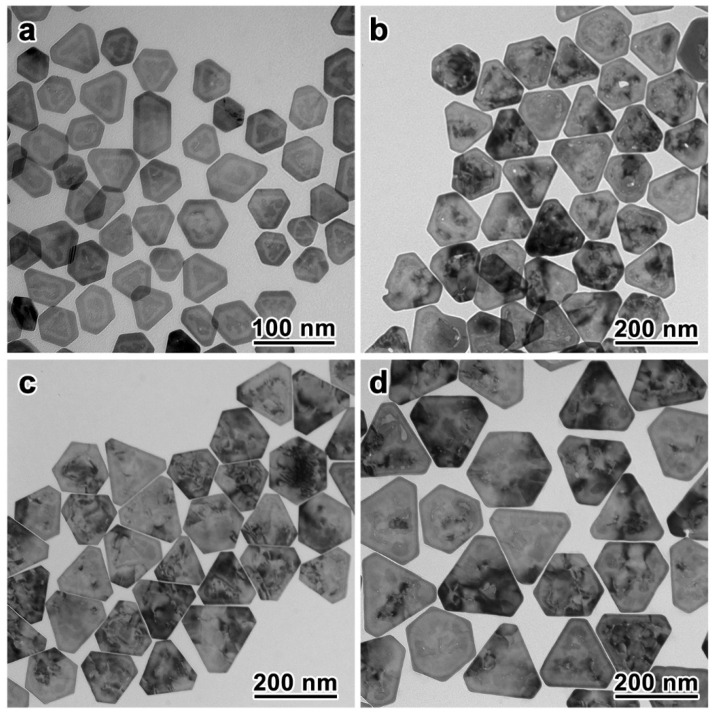
Synthesis of Au nanoplates with different sizes. (**a**–**d**) TEM images of the Au nanoplates with average edge lengths of 48, 115, 132, and 167 nm, respectively.

This synthesis is highly adaptable to afford Au nanoplates of different sizes for extended plasmonic properties in a wide range of the spectrum. Because the growth of the Au preferentially filled the inner cavity of the Au–Ag-alloy nanoframes, the final sizes of the Au nanoplates were consistent with those of the Au–Ag-alloy nanoframes. Therefore, the size of the Au nanoplates could be systematically tuned by employing Au–Ag-alloy nanoframes of different sizes, obtainable through the galvanic replacement of Ag nanoplates of specific sizes with HAuCl_4_, as the seeds. We demonstrated the synthesis of Au nanoplates with varying edge lengths in the range of 48 nm to 167 nm (Figure 3; more TEM images and size distributions can be found in Figures S4–S11). As confirmed by the TEM images, all these Au nanoplates were obtained with high morphological yield and size uniformity. Most of the nanoplates showed truncated triangular shapes with sharp edges after the seed-mediated growth.

**Figure 4:**
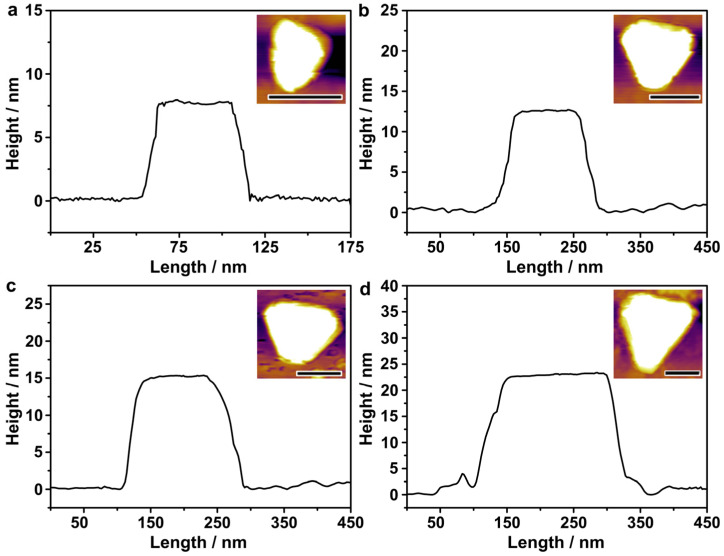
AFM characterizations of the thicknesses of the Au nanoplates. (**a**–**d**) Thickness profiles of the Au nanoplates with average edge lengths of 48, 115, 132, and 167 nm, respectively. The scale bars in the inset AFM images represent lengths of 100 nm.

The thicknesses of the Au nanoplates were measured by atomic-force microscopy (Figure 4). The Au nanoplates were first deposited on a clean silicon substrate. A tapping mode of the AFM was used to derive the thickness profiles of the Au nanoplates. The height difference between the Au nanoplate and the silicon substrate was defined as the thickness of the Au nanoplate. The thickness profiles were relatively smooth, suggesting uniform thicknesses across all the individual nanoplates. The thickness of the Au nanoplates with an average edge length of 48 nm was 7.5 nm, close to the value measured by TEM imaging (~8 nm). When increasing the edge length to 115, 132, and 167 nm, the average thickness increased to 12.5 nm, 15 nm, and 22.5 nm, respectively. This indicates that although the SO_3_^2−^ was strongly adsorbed on the Au {111} facets, the growth of the Au on both basal sides of the nanoplates was fully restrained, especially for those of large sizes that required extensive crystal growth. Nevertheless, the increase in the edge length was substantially quicker than the increase in the thickness, which highlights the role of SO_3_^2−^ in synthesizing Au nanoplates with small thicknesses and, therefore, high aspect ratios in the efficient shifting of the LSPR to long wavelengths.

**Figure 5:**
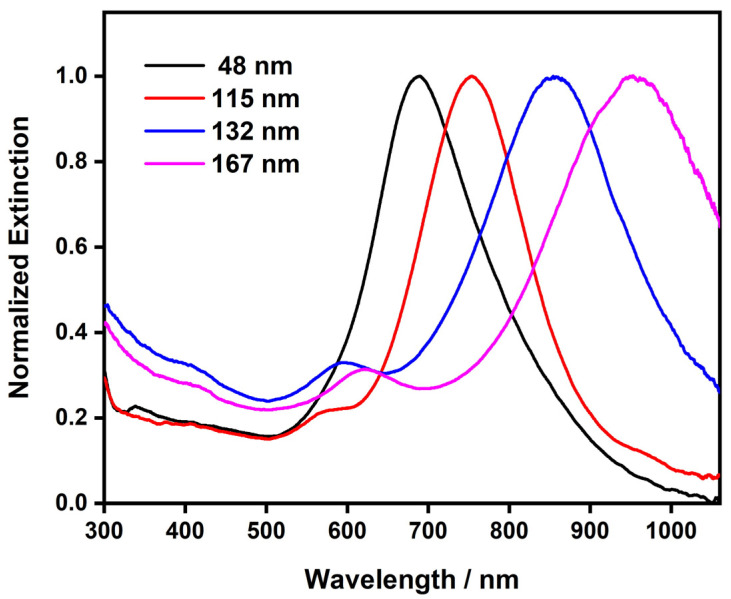
UV–Vis spectra of the Au nanoplates with different edge lengths.

The optical properties of plasmonic metal nanoplates depend greatly on their aspect ratios, which allowed us to effectively tune the LSPR band of the Au nanoplates by varying their edge lengths. Figure 5 shows the UV–Vis spectra of the Au nanoplates with different edge lengths. The in-plane dipolar LSPR band of the Au nanoplates with an average edge length of ~48 nm was at 685 nm. When increasing the edge length to 115 nm, 132 nm, and 167 nm, the dipolar LSPR band underwent a continuous redshift to 755 nm, 855 nm, and 955 nm, respectively. This observation revealed the clear dependence of the LSPR properties of the Au nanoplates on their size. In general, the in-plane dipolar LSPR bands underwent a continuous redshift in line with the increasing edge lengths of the Au nanoplates, which can be attributed to the increasing aspect ratios of the nanoplates. It is worth noting that a wet-chemical synthesis usually produces noble metal nanoplates with mixed triangular and hexagonal shapes, which was true in our synthesis. In addition, the nanoplates showed an edge-length distribution. Both may have affected the LSPR properties of the Au nanoplates. As a result, the dipolar LSPR bands of the Au nanoplates became broad, covering a range of wavelengths. Nevertheless, the LSPR bands were well defined and resolved. This suggests that the shape and edge-length uniformity of our materials was within a satisfactory threshold. This can be attributed to the well-designed seed-mediated synthesis, which enabled the simultaneous growth of the nanoplates at close rates. In addition, in-plane quadrupolar LSPR bands were also observed in the Au nanoplates with large edge lengths. They appeared at ~580 nm of the wavelength as a shoulder of the dipolar LSPR band. The appearance of the quadrupolar LSPR bands also indicated satisfactory uniformity in the shapes and sizes of the Au nanoplates obtained from our synthesis. With varying sizes, the Au nanoplates showed the LSPR band spanning from the visible to the near-infrared range of the spectrum. In particular, the LSPR bands of the Au nanoplates clearly covered the tissue optical window, which offers promise for many LSPR-based biological applications [4,9,54].

**Figure 6:**
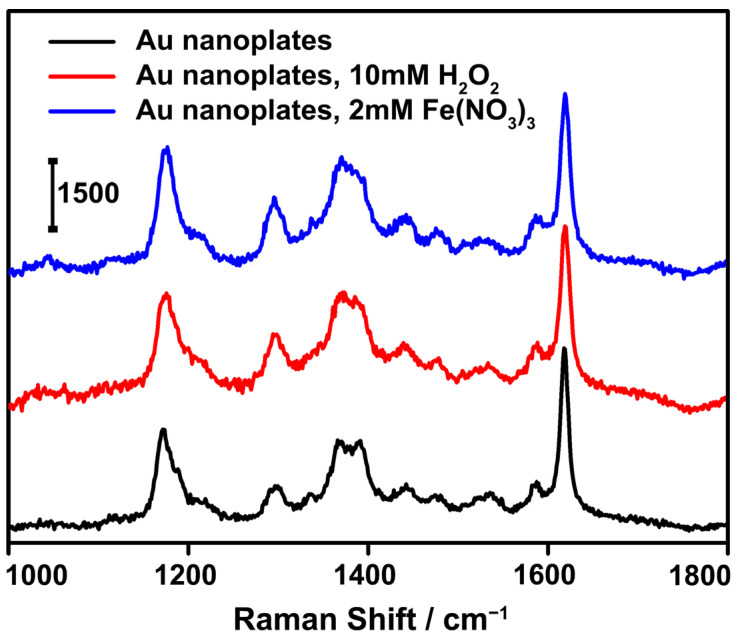
SERS analysis of crystal violet (concentration, 10^−6^ M) adsorbed on the substrates fabricated with the Au nanoplates. The SERS spectra in the presence of interfering species, i.e., 10 mM H_2_O_2_ and 2 mM Fe(NO_3_)_3_, are also listed for comparison.

We believe that Au nanoplates are excellent substrates for SERS applications, with clear advantages (Figure 6). First, the LSPR bands of the Au nanoplates are in the long-wavelength region of the spectrum, which is well apart from the electron interband transitions, leading to a high figure of merit and excellent Ag-like light absorption and scattering for near-field enhancement. Second, the nanoplates possess sharp edges and corners, which may serve as antennas to enable strong local electromagnetic fields [3,30,55]. Third, Au nanoplates are chemically inert and, thus, they can produce reliable Raman signals without interference, with many external chemical species. Therefore, we expected a highly sensitive and reliable SERS performance from the Au nanoplates. To demonstrate this, a substrate was prepared by drying Au nanoplates and a low concentration (10^−6^ M) of model organic molecules of interest for detection, i.e., CV, on a silicon wafer. Under laser irradiation with a wavelength of 633 nm, strong Raman signals characteristic of crystal-violet molecules were detected. The enhancement factor was calculated to be ~1.8 × 10^4^ (Supporting Information). When oxidative species, such as H_2_O_2_ and Fe^3+^, were introduced onto the substrate, no noticeable changes in the Raman signals of the crystal violet were detected. The intensities of the SERS signals were almost identical to those obtained in the absence of the interfering species. No obvious structural change in the Au nanoplates was detected by the TEM imaging, which accounted for the high SERS stability (Figures S12 and S13). These results suggest the high sensitivity and excellent reliability of the SERS detection of molecules of interest when using Au nanoplates as the substrates. We expect these Au nanoplates to be particularly useful in practical SERS applications, in which interfering species are inevitably present.

## 4. Conclusions

In summary, we demonstrated a robust method for synthesizing thin Au nanoplates with tunable sizes and excellent surface-plasmon-resonance properties, starting from Au–Ag-alloy nanoframe seeds. The sulfite ligand played a pivotal role in this synthesis. First, its coordination with the Au(III) salt reduced the reduction potential, leading to the controlled crystal growth of Au on the Au–Ag-alloy-nanoframe seeds. Second, the sulfite adsorbed strongly on the basal Au {111} facets, which not only guided the formation of the plate-like crystal shape but also restrained the crystal growth on the basal sides of the Au nanoplates, leading to significantly reduced thicknesses compared with those reported in the literature. The Au nanoplates can be as thin as 7.5 nm, although their thickness may increase with the edge length. The edge length can be customized in the range of 48–167 nm, leading to tunable surface-plasmon-resonance bands in the range of 600–1000 nm. Due to their high stability and remarkable optical properties, the Au nanoplates developed in this work are particularly useful in many LSPR-based applications, in which interfering species are usually involved. As a demonstration of this, Au nanoplates showed superior performance in the SERS detection of molecules of interest in the presence of Fe^3+^ and H_2_O_2_. We believe this strategy opens a new route for the synthesis of Au nanoplates with controlled LSPR properties for many biological, analytical, and catalytic applications.

## Data Availability

The data presented in this study are available on request from the corresponding author.

## Supplementary Materials

The following supporting information can be downloaded at: https://www.mdpi.com/article/10.3390/nano13040711/s1. Figure S1: Characterization of Ag nanoplates. (a, b) TEM images of Ag nanoplates. (c) Size distribution of Ag nanoplates. (d) Thickness histogram of Ag nanoplates. The average size of the Ag nanoplates was measured as ~45 nm. The average thickness was calculated as ~6.8 nm. Figure S2: Low-magnification TEM image of the Au–Ag-alloy nanoframes. Figure S3: EDS mapping of the Au–Ag-alloy nanoframe. Figure S4: Low-magnification TEM image of the Au nanoplates (edge length, ~48 nm). Figure S5: Size histogram of the Au nanoplates (edge length, ~48 nm). Figure S6: Low-magnification TEM image of the Au nanoplates (edge length, ~115 nm). Figure S7: Size histogram of the Au nanoplates (edge length, ~115 nm). Figure S8: Low-magnification TEM image of the Au nanoplates (edge length, ~132 nm). Figure S9: Size histogram of the Au nanoplates (edge length, ~132 nm). Figure S10: Low-magnification TEM image of the Au nanoplates (edge length, ~167 nm). Figure S11: Size distribution of the Au nanoplates (edge length, ~167 nm). Figure S12: TEM image of the Au nanoplates after treatment in H_2_O_2_ (10 mM) for 10 h. Figure S13: TEM image of the Au nanoplates after treatment in Fe(NO_3_)_3_ (2 mM) for 10 h. Figure S14: SERS of a crystal-violet solution (concentration, 10^−5^ M) without Au nanoplates (signal acquisition time, 30 s).
