# Supplementary material for: Seed-Mediated Synthesis of Thin Gold Nanoplates with Tunable Edge Lengths and Optical Properties
Source: Nanomaterials (Basel). 2023 Feb 13;13(4):711. doi: 10.3390/nano13040711 (PMC9961956; doi:10.3390/nano13040711)
Supplement: Supplementary file 1 [file nanomaterials-13-00711-s001.zip › nanomaterials-2187099-supplementary.pdf]

nanomaterials

Supporting Materials

Seed-Mediated Synthesis of Thin Gold Nanoplates with Tunable Edge Lengths and Optical Properties

Zhun Qiao †, Xinyu Wei †, Hongpo Liu, Kai Liu * and Chuanbo Gao *

Frontier Institute of Science and Technology, Xi'an Jiaotong University, Xi'an 710054, China
* Correspondence: liu.k.n@xjtu.edu.cn (K.L.); gaochuanbo@mail.xjtu.edu.cn (C.G.)
† These authors contributed equally to this work.

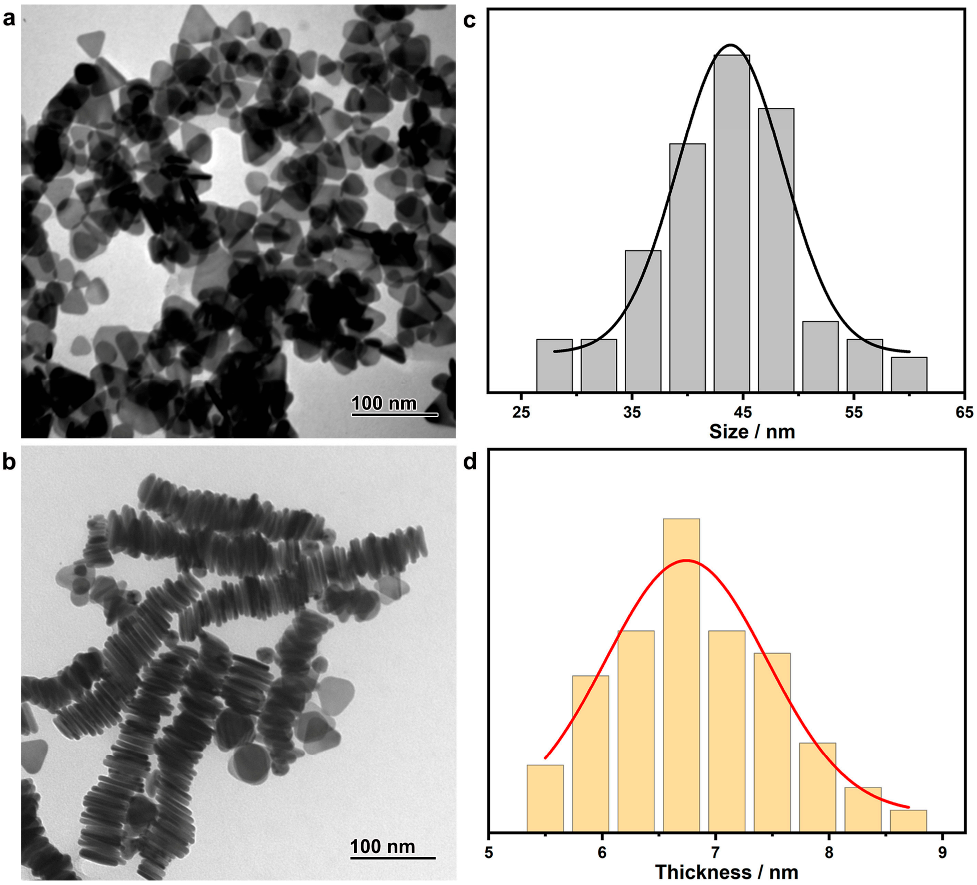

Figure S1. Characterization of Ag nanoplates. (**a**,**b**) TEM images of Ag nanoplates. (**c**) Size distribution of Ag nanoplates. (**d**) Thickness histogram of Ag nanoplates. The average size of the Ag nanoplates was measured as ~45 nm. The average thickness was calculated as ~6.8 nm.

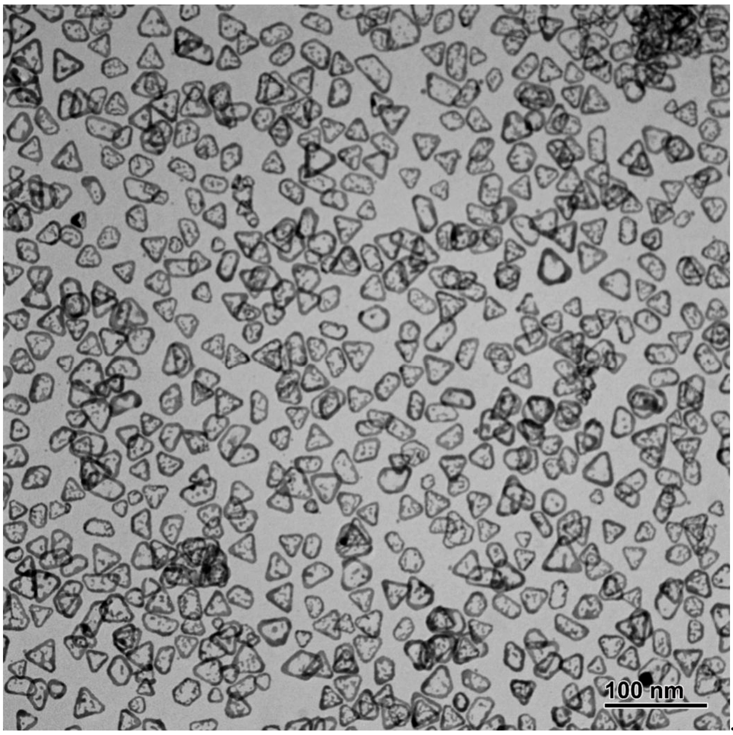

Figure S2. Low-magnification TEM image of the Au-Ag-alloy nanoframes.

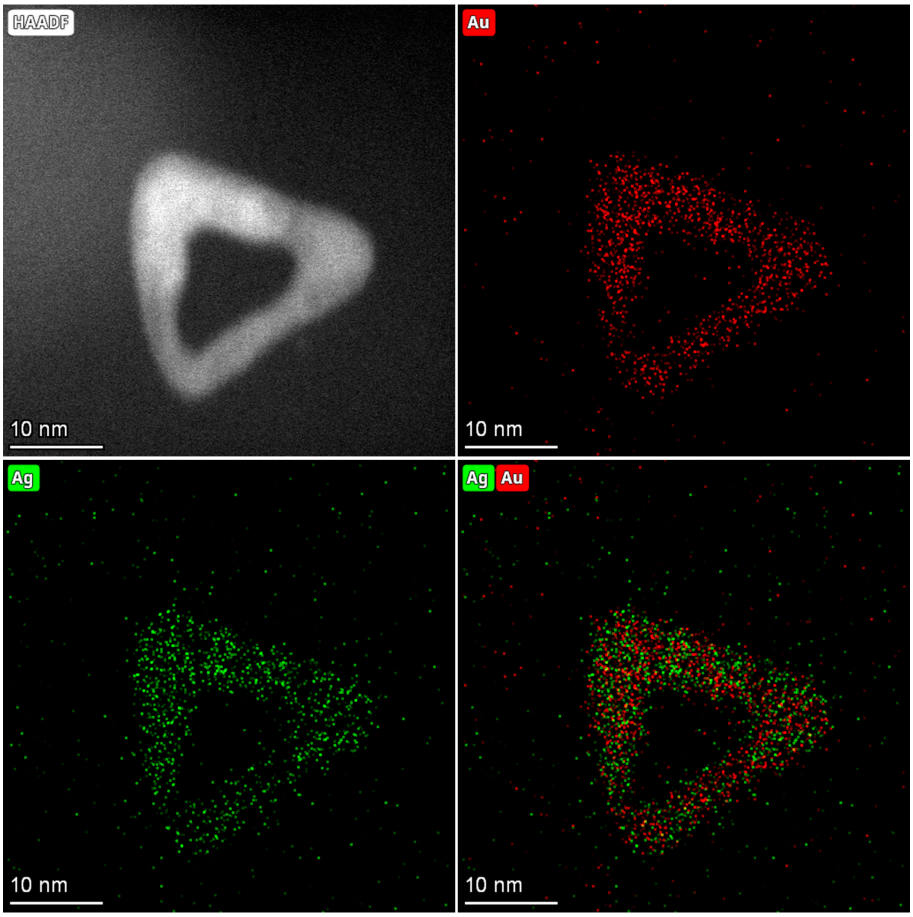

Figure S3. EDS mapping of the Au-Ag-alloy nanoframe.

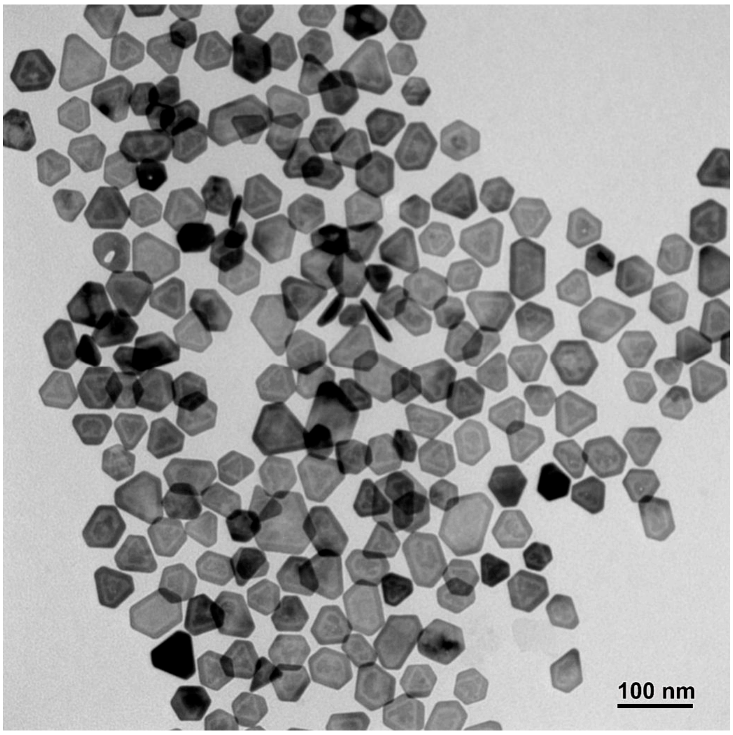

Figure S4. Low-magnification TEM image of the Au nanoplates (edge length, ~48 nm).

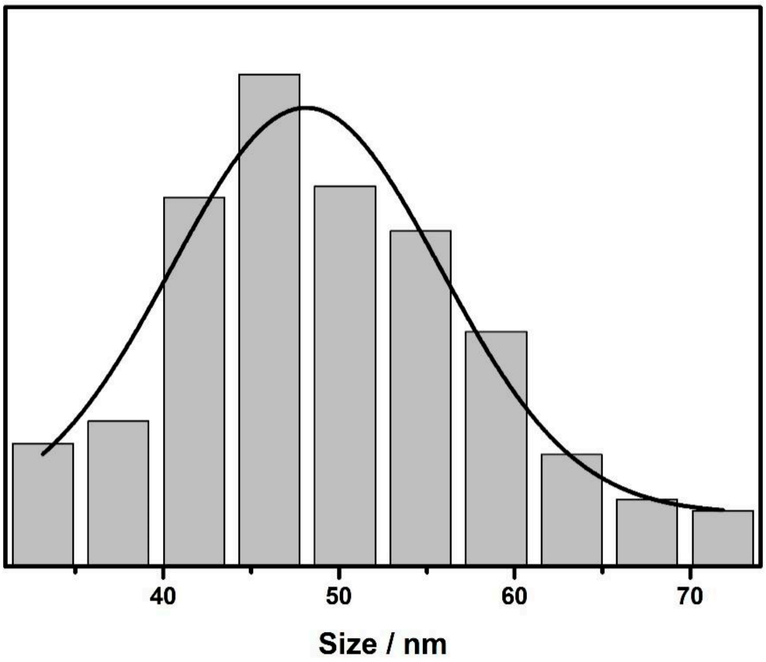

Figure S5. Size histogram of the Au nanoplates (edge length, ~48 nm)..

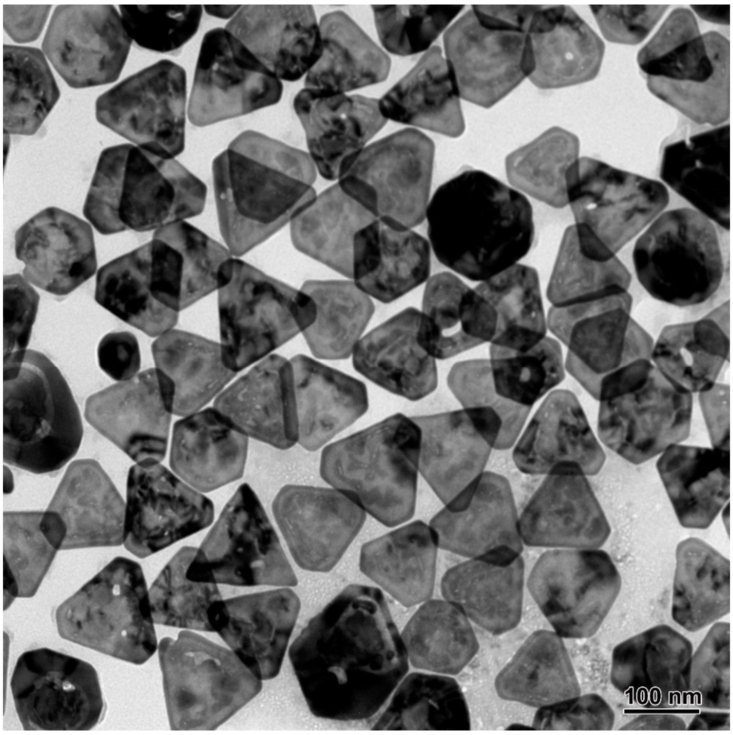

Figure S6. Low-magnification TEM image of the Au nanoplates (edge length, ~115 nm).

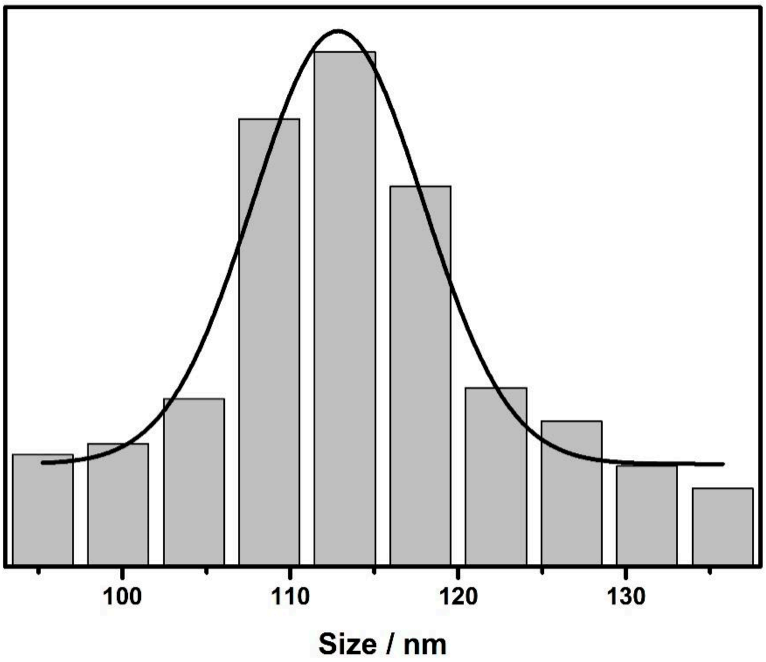

Figure S7. Size histogram of the Au nanoplates (edge length, ~115 nm).

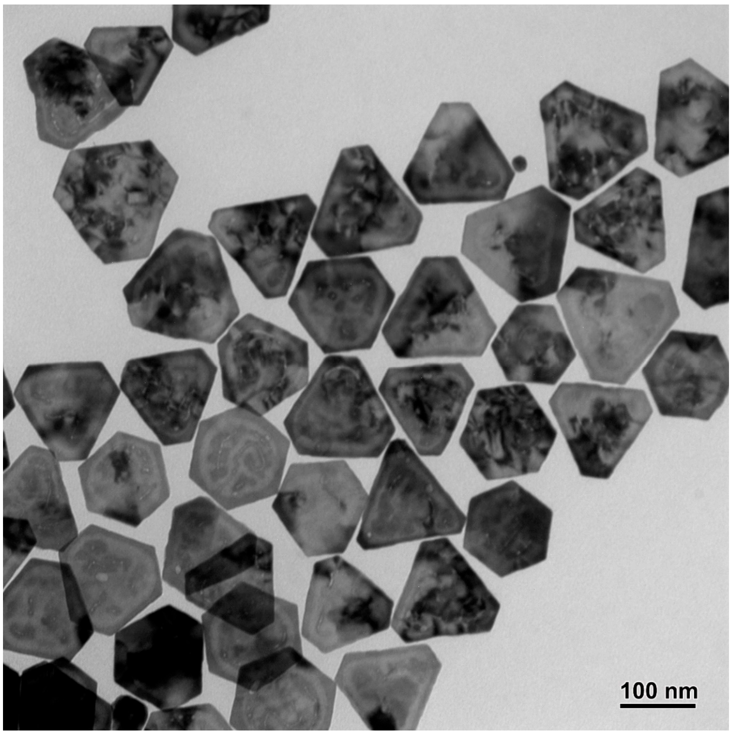

Figure S8. Low-magnification TEM image of the Au nanoplates (edge length, ~132 nm).

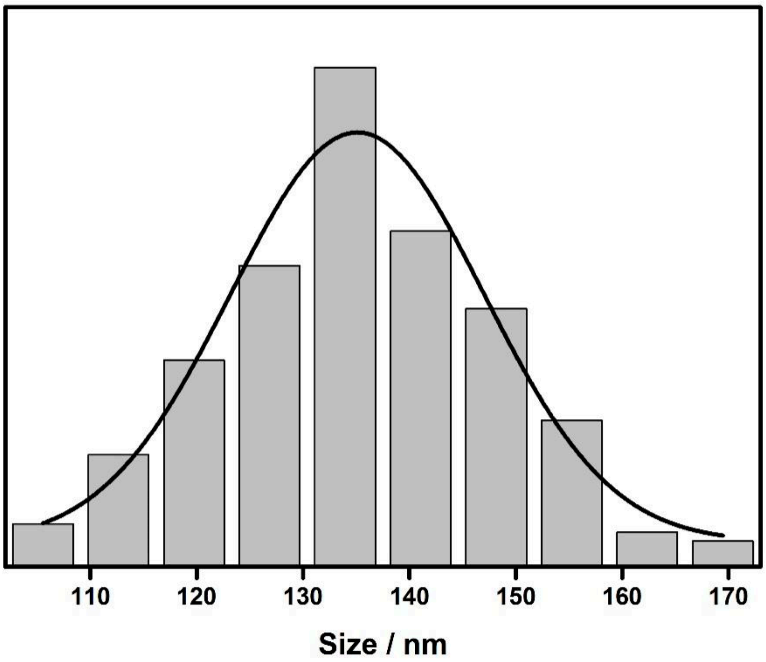

Figure S9. Size histogram of the Au nanoplates (edge length, ~132 nm)..

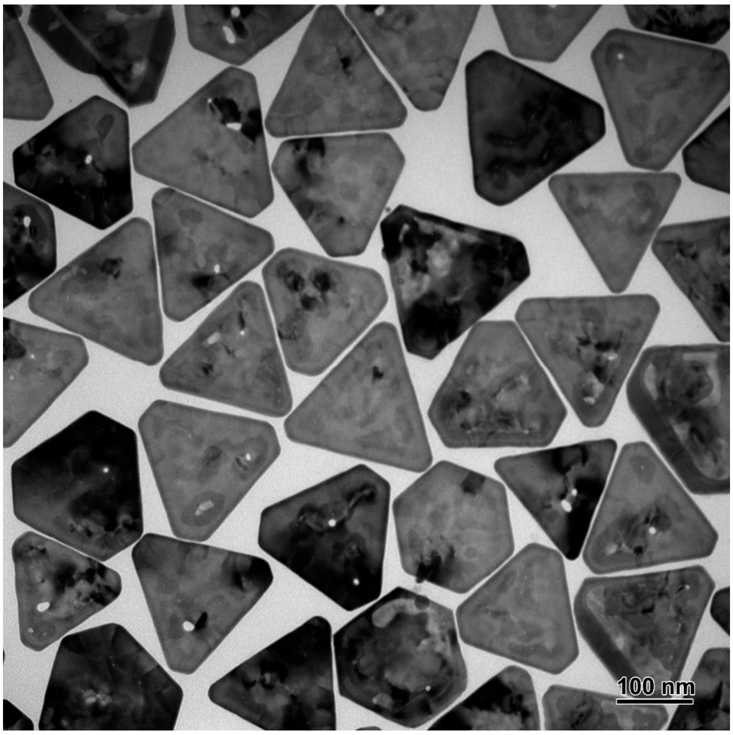

Figure S10. Low-magnification TEM image of the Au nanoplates (edge length, ~167 nm).

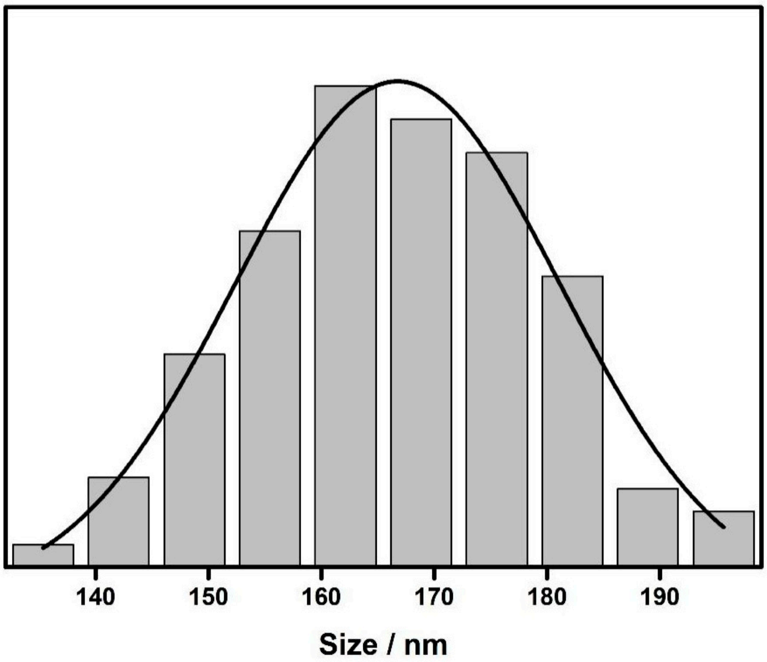

Figure S11. Size distribution of the Au nanoplates (edge length, ~167 nm).

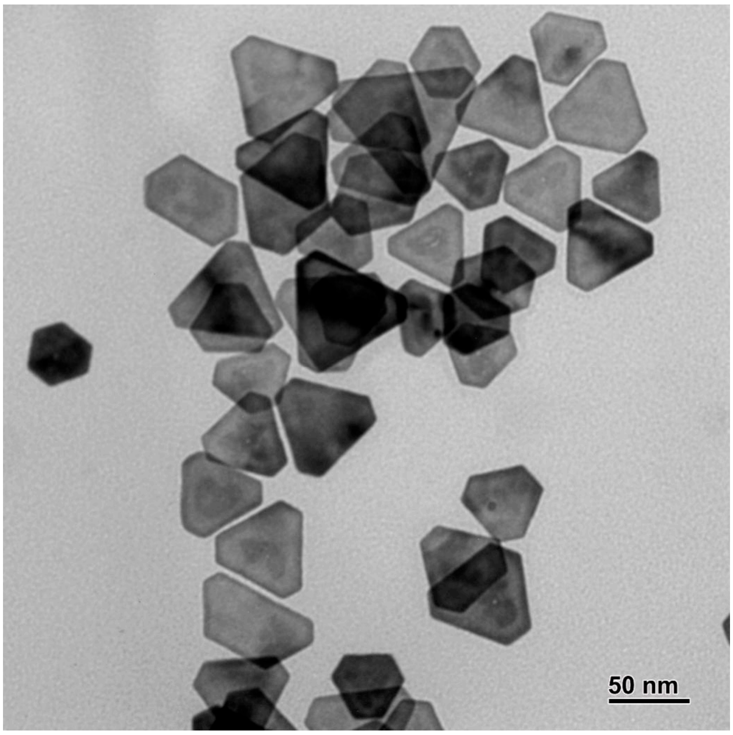

Figure S12. TEM image of the Au nanoplates after treatment in H_2O_2 (10 mM) for 10 h.

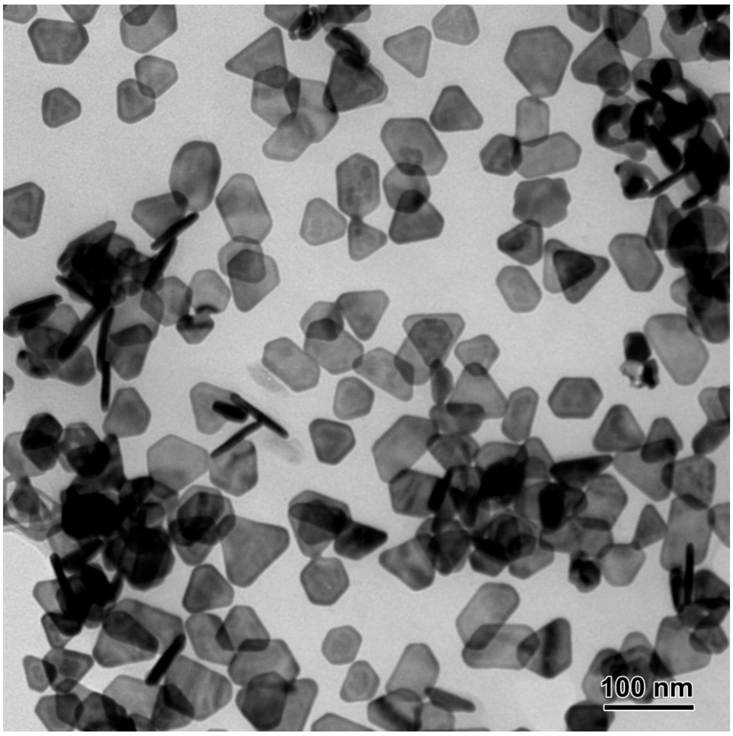

Figure S13. TEM image of the Au nanoplates after treatment in $Fe(NO_3)_3$ (2 mM) for 10 h.

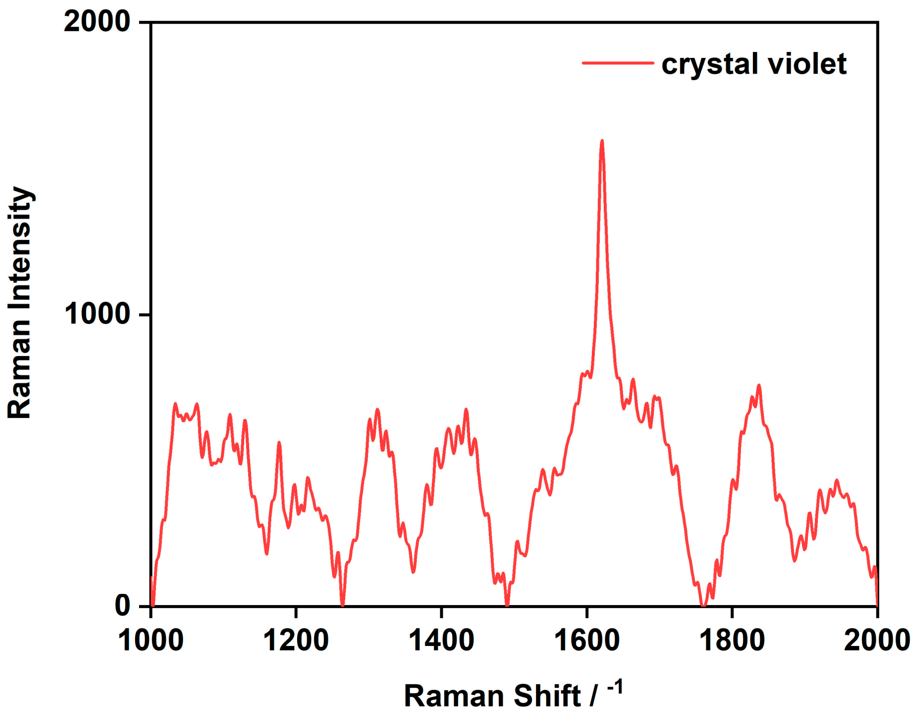

Figure S14. SERS of a crystal violet solution (concentration, 10^{-5} M) without Au nanoplates (signal acquisition time, 30 s.).

Calculation of the enhancement factor (EF). The enhancement factor of Au nanoplates (edge length, ~48 nm) was estimated using the following equation:

$$EF = \left(\frac{I_{surface}}{N_{surface}}\right)/\left(\frac{I_{solution}}{N_{solution}}\right)$$

Here, $I_{surface}$ and $N_{surface}$ are the Raman intensity probed from a single Au nanoplate and the number of crystal violet molecules (concentration, 10^{-6} M) absorbed on a single Au nanoplate. $I_{solution}$ and $N_{solution}$ are the Raman intensity probed from a solution of crystal violet (concentration, 10^{-5} M) in a capillary and the number of crystal violet molecules in the focus of the laser beam.

The Raman intensity $I_{surface}$ was 6793 counts at the peak of 1618 cm^{-1} (laser power density, 51.67 kW cm^{-2}; accumulation time, 10 s). $I_{solution}$ was measured to be 812 counts (laser power density, 39.8 kW cm^{-2}; accumulation time, 30s), which is equivalent to 351 counts under the laser irradiation of 51.7 kW cm^{-2}. $N_{solution}$ was estimated using the following equation:

$$N_{solution} = 6.02 \times 10^{23}\, mol^{-1} \times 10^{-5} mol/L \times \left[\pi \left(\frac{d}{2}\right)^2 \times H\right] = 10 = 1.8 \times 10^7$$

where d is the diameter of the light spot and can be calculated to be 3.1 μm; H is the effective depth of focus and can be estimated to be 400 μm.

$N_{surface}$ can be estimated as:

$$N_{surface} = S_0/S_{Si} \times S_{coverage} \times N_{CV}$$

where S_0 is the surface area of the light spot,

$$S_0 = \pi \times 3.1\ \mu m^2$$

S_{Si} is the surface area of the substrate (S_{Si} = 6.4 × 10^{13} nm^2).

$S_{coverage}$ is the coverage of the Au nanoplates in the substrate,

$$S_{coverage} = \frac{N_{Au}}{N_{single}} \times \frac{S_{Au}}{S_{Si}} \times \eta$$

where N_{Au} is the number Au atoms in all the Au nanoplates from a single batch of the synthesis assuming a yield of ~80%,

$$N_{Au} = 0.25\ mol/L \times 4 \times 10^{-5}\ L \times 0.6\ mL/8\ mL\ /2\ mL \times 0.025\ mL \times 6.02 \times 10^{23}\ mol^{-1} \times 80\% = 4.5 \times 10^{15}$$

N_{single} is the number of Au atoms in a single Au nanoplate,

$$N_{single} = 48/0.2355 \times (48/0.2355 + 1)/2 \times 6.8/0.2039 = 7.0 \times 10^5$$

S_{Au} is the expose surface area of an Au nanoplate,

$$S_{Au} = \pi \times 48^2/4$$

η is a coefficient reflecting the effect of nanoplate aggregation on the substrate; $\eta = 0.5$.

Thus, $S_{coverage} = 0.06$

In the experiment, 25 μL of an aqueous crystal violet solution (10^{-6} M) was dried onto the substrate and dipped 3 times in water to remove weakly absorbed molecules. About ~6.8 % of the crystal violet molecules remained after dipping. Thus,

$$N_{CV} = 25 \times 10^{-6} L \times 10^{-6} mol/L \times 6.02 \times 10^{23} mol^{-1} \times 6.8\% = 1.0 \times 10^{12}$$

Therefore,

$$N_{surface} = 1.9 \times 10^4$$

$$EF = \left(\frac{Isurface}{Nsurface}\right)/\left(\frac{Isolution}{Nsolution}\right) \approx 1.8 \times 10^4$$
